# Development of a Controlled-Ventilation Box for Modified-Atmosphere Storage of Fresh Produce

**DOI:** 10.3390/foods10122965

**Published:** 2021-12-02

**Authors:** Nandita Keshri, Ingo Truppel, Manfred Linke, Martin Geyer, Cornelia Weltzien, Pramod Mahajan

**Affiliations:** 1 Department of Horticultural Engineering, Leibniz Institute for Agricultural Engineering and Bioeconomy (ATB), Max-Eyth-Allee 100, 14469 Potsdam, Germany; nkeshri@atb-potsdam.de (N.K.); itruppel@atb-potsdam.de (I.T.); mlinke48@yahoo.de (M.L.); mgeyer@atb-potsdam.de (M.G.); 2 Agromechatronics–Sensor-Based Process Management in Agriculture, Technische Universität Berlin, Straße des 17, Juni 135, 10623 Berlin, Germany; CWeltzien@atb-potsdam.de

**Keywords:** wireless sensor system, forced airflow, mass transfer coefficient, metal tube, real-time, O_2_ and CO_2_ concentration

## Abstract

Adjusting beneficial gas concentrations in real time in response to changing storage conditions is important for fresh produce, especially throughout the supply chain when temperature abuse occurs frequently. In this study, a controlled-ventilated box for bulk transportation of fresh produce was demonstrated and tested under variable temperatures. The presented system comprised a rigid container with a miniature blower installed in the opening of its wall for facilitating the gas exchange and an additional wall opening with a metal tube protruding into the inner container’s space. The in-package atmosphere was formed by the balance between the respiratory activity of the produce and the influx of fresh air through the wall openings, regulated by switching the blower ON or OFF. The mass transfer coefficient for metal tubes of different dimensions was measured under modified atmosphere featuring 15% CO_2_ and 5% O_2_ at 10 °C. The addition of an air blower increased the mass transfer coefficient by at least 100 times. A further storage trial with cherries was successfully performed at 10 °C and 20 °C. The demonstrated trial featured some significant inputs to increase the knowledge about better storage of fresh produce throughout the supply chain and storage.

## 1. Introduction

Fresh fruit and vegetables (F&V) are highly perishable, making their supply a race against the clock for growers, shippers, and retailers to maintain their quality and reduce losses. Postharvest F&V losses are typically the highest among all food categories, varying in most industrialized and developing regions between 45% and 55% [1]. To reduce these losses, producers and handlers must first understand the biological and environmental factors involved in deterioration, and second, use appropriate postharvest storage techniques that delay degradation and maintain the best possible quality.

Temperature, optimum O_2_ and CO_2_ concentrations and respiration rate of F&V are the major factors that influence their postharvest life [2,3]. For decades, modified atmosphere (MA) and cold storage technologies have remained the most widely used transportation technologies for fresh produce worldwide. MA can be achieved by packing fresh produce in permeable films that can modify the O_2_ and CO_2_ concentrations mostly due to respiration of the produce and permeability of the packaging material itself, hence is relatively inexpensive. The major weakness of the MA approach is insufficient atmosphere control, especially in the situation when a temperature shift changes the produce respiration rate. Lack of interactive adaptation to external (temperature) and internal (produce amount and respiratory activity) factors is a common drawback of most MA systems [4]. Temperature abuse in the supply chain and the retail display is one concern regarding the safety of fresh produce. Higher than optimum temperature increases the respiration rate which in turn expedites CO_2_ release by stored produce and ultimately leads to anaerobic condition inside the package, thereby shortening its storage life [5]. This presents a major obstacle to applying MA technologies in the supply chain. Complementing temperature control with optimal atmosphere composition (product-specific beneficial levels, typically reduced O_2_ and elevated CO_2_) can additionally extend the produce life by 25% or even more [6].

Several studies have been performed to improve the traditional MA system. One example is tuning gas permeation by using micro-perforations or macro-perforations on packages [7,8] or perforation tubes inserted in an otherwise impermeable rigid box [9]. Several further studies were conducted to investigate the effect of tube dimensions, length (L) and diameter (D), and temperature on gas exchange through perforation tubes [9,10,11,12,13]. Silva, Chau, Brecht, and Sargent [9] studied the effect of tube dimensions and temperature (1.5 °C, 7 °C, and 19 °C) on gas exchange and found out that temperature has no significant effect on the gas mass transfer coefficient of O_2_ (*K*_O_2__) and CO_2_ (*K*_CO_2__). Fonseca, Oliveira, Lino, Brecht, and Chau [11] modelled the relationship between L and D of the tube and mass transfer coefficients (Equations (1) and (2)).

(1) $$K_{O_{2}}={\;a\;D}^{b}{\;L}^{-c}\;$$

(2) $$K_{{\mathrm{CO}}_{2}}=\beta \;\;K_{O_{2}}$$

where *K*_O_2__, *K*_CO_2__ are measured in m^3^ s^−1^ and a (model parameter)—in m^3-b-c^ s^−1^, D and L are diameter and length of the tube in (m), *β* is the permeability ratio (dimensionless), and model parameters b and c are dimensionless.

The study reported that mass transfer coefficients *K*_O_2__ and *K*_CO_2__ increase with the increase in tube D and decrease with the increasing tube L. It was also reported that the effect of tube diameter is more pronounced for small-length tubes, while the effect of tube length becomes more important as the tube diameter increases. The studies mentioned above were conducted for small-sized boxes/packages (max. 3.78 L) and tube sizes varying from 5 mm to 40 mm in length and from 6.5 mm to 17 mm in diameter. Due to their passive mode of air exchange (diffusion) through a tube between the inside and outside atmosphere of the storage container, it is expected that tubes alone cannot maintain the desired gas composition inside the abovementioned storage boxes/packages. Therefore, such boxes, when scaled up and used for bulk storage and transportation of F&V, are more likely to fail in maintaining the desired gas composition inside a storage box, especially when subjected to fluctuating temperatures (high temperatures) often encountered in the supply chain [14,15]. Therefore, it is necessary to design a system that is capable of actively maintaining the desired gas composition based on real-time changes in temperature and the resulting changes in respiration rates of the stored produce.

Jo et al. [16] developed a concept of dynamically controlling gas composition in an MA package (0.32 m × 0.23 m × 0.18 m) by installing gas sensors, a diffusion tube, and a ball valve attached to the tube. In this system, the package was assumed to be hermetically closed, MA was created by the respiration of the stored produce. Even though such a system satisfactorily maintained the desired gas composition inside the MA package at a low temperature (10 °C), it is uncertain whether the same results can be achieved at higher temperatures, such as 20 °C. The reason is that with increasing temperature, respiration will increase and the atmosphere inside the package will change rapidly. Therefore, either the tube dimensions need to be changed or the air exchange needs to be expedited. A further advancement by Jo et al. [17] was to introduce an air pump to actively flush the MA package. The active flushing system was shown to be more prompt in its response to deviating atmospheric conditions even at a higher temperature (20 °C). However, mass transfer modelling and studying the effect of tube D and L on the mass transfer coefficient under a forced convective flow were not performed. In a further study, Lee et al. [18] demonstrated an MA control system with a gas diffusion tube operating adaptively in response to O_2_ concentrations, which changed with respiration activity depending on temperature. In this study, the authors applied hypobaric pressure in order to achieve rapid pulldown of the O_2_ concentration inside the storage container. However, even with the application of the initial hypobaric pressure, it took about 3.4 d to attain the target gas concentration inside the storage box. Time as long as 3.4 d, however, could be the end of shelf life of some F&V.

The aim of this study was to develop a method to indirectly control CO_2_ and O_2_ concentrations in a controlled-ventilation storage box as a function of fluctuating temperature. This hypothesis was tested with the help of a thin and long metal tube, which largely prevents air from entering the box but facilitates air exchange when a miniature air blower is switched ON as a function of temperature. In this study, the time the air blower was switched ON was regulated based on the actual storage temperature. The developed system was validated for sweet cherries at 10 °C and 20 °C. 

## 2. Materials and Methods

### 2.1. Experimental Setup 

**Figure 1:**
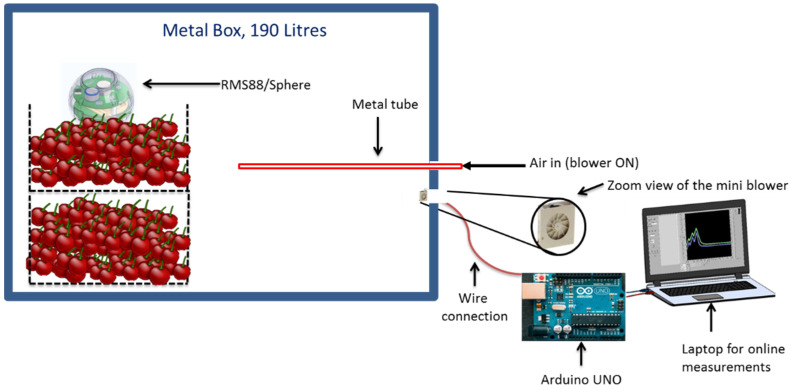
The experimental setup used for evaluating the effect of forced airflow on the mass transfer coefficient in a 190 L metal box connected to a metal tube (different diameters and lengths). A 12 mm × 12 mm × 3 mm blower with the flow rate of 2.7 L min^−1^ operated by an Arduino UNO was installed in an opening of the box to facilitate forced airflow. Changes in the O_2_ and CO_2_ concentrations were measured using a modular sensor system (RMS88) that transferred data in real time to the gassensor software on a laptop.

Airtight 190 L metal boxes kept inside a walk-in cold room were used for the experiments. Figure 1 shows the experimental setup. A refrigeration system (Frigotech GmbH, Landsberg, Germany) was used to maintain the desired temperatures inside the cold room. A metal tube was inserted through an opening on a side wall of metal boxes. In the second opening of the same wall, inside the boxes, a 12 mm × 12 mm × 3 mm mini air blower (Sunonwealth Electric Machine Industry, Kaohsiung, Taiwan) was installed, and when switched ON, brought the air from the outer atmosphere (flow rate of 2.67 L min^−1^; free flow condition) to the metal box. This blower was selected due to its comparatively small airflow rate and low energy consumption and operated using an Arduino Uno (Arduino.cc) for switching it ON/OFF using the Arduino software interface on a laptop. The duct in which the blower was mounted was a 3D-printed ABS (acrylonitrile butadiene styrene) plastic tube of 16 mm diameter and 50 mm length. In this study, the pressure drop contributions from the duct in which the blower was mounted were neglected due to the low length-to-diameter ratio as compared to the tubes used for mass transfer. This was because the Hagen–Poiseuille equation does not hold true for the duct due to the length-to-diameter ratio being less than Re/48, where Re is the Reynolds number [19].

A gas measurement sensor (RMS88) as described by Keshri et al. [20] was also placed inside the metal boxes. It is a small-sized (diameter: 88 mm) modular system that consists of a fluorescence-based optical O_2_ sensor (measurement range of 0–25%, resolution: 0.01%, accuracy: 2% of the full scale) and a nondispersive infrared CO_2_ sensor (measurement range of 0–200,000 ppm, resolution: 10 ppm, accuracy: 70 ppm (5% of the reading)) (SST Sensing Ltd., Coatbridge, UK). Moreover, RMS88 additionally measures relative humidity (RH) and temperature. The software for using RMS88 called Gassensor was developed in-house (LabView, National Instruments Corporation, Austin, TX, USA) and used for receiving the wireless data for gas concentrations inside the boxes. PCs recognize RMS88 as a serial device and a simple dialog menu allows full control of RMS88 via the serial terminal. As soon as RMS88 is activated, it starts real-time measurement and recording, wirelessly transferring O_2_, CO_2_, temperature, and RH data, and is immediately placed inside the storage chamber. Based on real-time data, the blower ON frequency was adjusted manually in Arduino Uno. Two RMS88 sensors were placed inside the box and a gas analyzer (PBI Dansensor, Ringsted, Denmark) was used to measure gas concentrations at different locations of the box to confirm that there was no gas stratification and the O_2_ and CO_2_ concentrations were homogenous.

### 2.2. Measurement of the Mass Transfer Coefficient

With all the combinations of different tube dimensions (D: 2 mm, 4 mm, and 6 mm; L: 50 mm, 250 mm, and 500 mm), using the full factorial design, nine unique experiments were performed at the constant temperature of 10 °C using the experimental setup as shown in Figure 1 (without cherries). For each combination of tube D and L, two replicate experimental runs were performed, giving a total of 18 experimental runs. Metal boxes were completely closed and initially flushed with modified gas containing 5% O_2_ and 15% CO_2_ using a gas mixture and a control system provided by Frigotec GmbH. Once the boxes were completely flushed, the metal tubes were opened from the outside for 4 h, followed by switching ON the mini blower continuously for the rest of the measurement period. 

Data collected by RMS88 was used to estimate the diffusion rate due to diffusion (blower OFF) and mass transfer coefficients due to forced airflow (blower ON) for O_2_ (*K*_O_2__) and CO_2_ (*K*_CO_2__) using a statistical method. *K*__O2__ and *K*_CO_2__ were estimated by fitting the data into a lumped capacitance model suggested by Emond, Castaigne, Desilets, and Toupin [10] when there is no produce in the package. The model was fitted to the experimental data using the nonlinear regression method. Assuming that there is 21% O_2_ and 0.04% CO_2_ in the surrounding atmosphere of the storage boxes, Equations (3) and (4) were used to estimate the *K*_O_2__ and *K*_CO_2__ coefficients:

(3) $$y_{O_{2},t}=21+\left(y_{O_{2},\mathrm{int}}\;-\;21\right)\;exp\left(-\frac{K_{O_{2}}}{V}t\right)$$

(4) $$y_{{\mathrm{CO}}_{2},t}=0.04+\left(y_{{\mathrm{CO}}_{2},\mathrm{int}}-\;0.04\right)\;exp\left(-\frac{K_{{\mathrm{CO}}_{2}}}{V}t\right)$$

where *y_t_* is the volumetric concentration of the respective gases (%) within the container at time *t* (s), *y*_ini_ is the initial volumetric gas concentration (%) within the container, *V* is the volume of the container (m^3^), and *K*_O_2__ and *K*_CO_2__ are the diffusion rate/mass transfer coefficients (m^3^ s^−1^).

### 2.3. Storage Trial with Sweet Cherries

The storage experiment was performed with sweet cherries freshly harvested in July from a farm in Marquardt, Germany. Sweet cherries (25 kg) were stored in a metal box at varying temperatures (10 °C for 114 h and 20 °C for 21 h) (Figure 1). The reason to increase the temperature in this study was to test the developed box at a higher temperature. Such temperature variations are often encountered during the supply of fresh produce. This study experimentally evaluated a box that compensates the increase in temperature by increasing the blower ON time. A metal tube of 4 mm D and 250 mm L was chosen for this experiment. As per Mitcham et al. (2002), the optimal window for successful MA storage of sweet cherries is 3–10% O_2_ and 10–15% CO_2_; hence, the box was initially flushed with 6.5% O_2_ and 12.5% CO_2_, the average of the recommended gas concentration values taken as the initial setpoints. The initial flushing was performed using a gas mixture and a control system (Frigotec GmbH, Landsburg, Germany). The metal box was isolated from the control system after flushing. After a short period of time, the blower was switched ON to facilitate forced airflow through the tube between the outer atmosphere and the inside of the storage box. The blower ON frequency, which is the blower operating time in minutes per hour, was initially set to 4 min h^−1^. The blower ON frequency was adjusted manually based on the threshold criteria of ±0.5% deviation from the setpoint for O_2_ and CO_2_ concentrations and temperature assuming the respiration rate of fresh produce doubles for every 10 °C change [3].

### 2.4. Statistical Analysis

Experiments for the measurement of the diffusion rate and the mass transfer coefficient were performed in duplicates. *K*_O_2__ and *K*_CO_2__ were estimated by fitting Equations (3) and (4) to the experimental data by means of nonlinear regression using the Statistica software (StatSoft Inc., Tulsa, OK, USA) and the solver function in Microsoft Excel (Microsoft Corporation, Redmond, WA, USA). The *K*_O_2__ and *K*_CO_2__ values obtained were submitted to the two-factor analysis of variance (ANOVA) test with significance set at *p* < 0.05 using Microsoft Excel (Microsoft Corporation, Redmond, WA, USA). The results were presented as the means ± standard deviation.

## 3. Results

### 3.1. Gas Exchange Due to Forced Airflow

**Figure 2:**
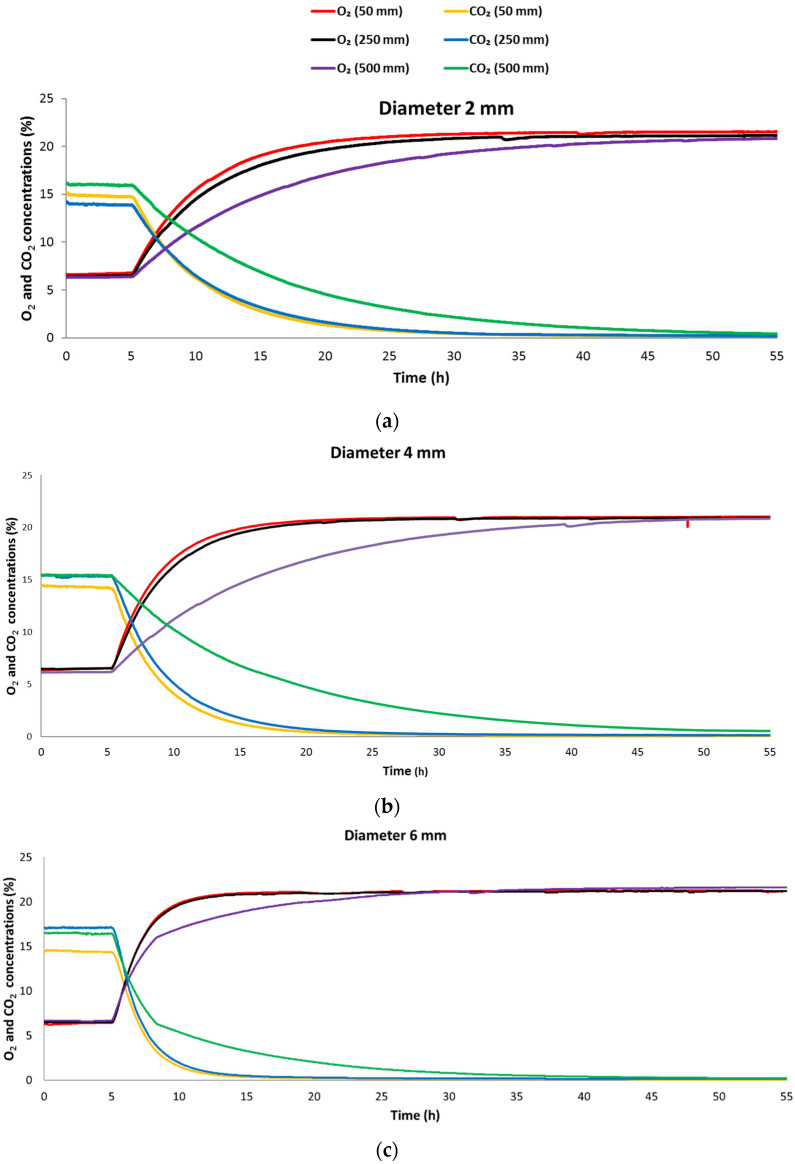
Data collected in real time for changes in the concentrations of O_2_ and CO_2_ over time in an empty metal box flushed with 15% O_2_ and 5% CO_2_ and fitted with one metal tube (combinations of L of 50 mm, 250 mm, and 500 mm and D of (**a**) 2 mm, (**b**) 4 mm, and (**c**) 6 mm; nine unique experiments) and a mini blower continuously ON after 4 h of flushing.

Figure 2a–c shows the experimental data for the O_2_ and CO_2_ concentrations inside the metal boxes during the blower OFF and ON modes. Initial slow changes in gas concentrations were observed when the blower was OFF (initial 4 h) and gas exchange occurred through the metal tube and the air blower by diffusion only. Switching ON the blower (forced airflow) at the 5th hour resulted in a sudden increase in gas exchange. The gas equilibrium was not completely achieved in the measuring period; therefore, it was expressed in terms of a dimensionless parameter as follows:

(5) $$C_{eq}^{*}=\frac{\left(C_{eq}-C_{ext}\right)}{\left(C_{\mathrm{ini}}-C_{\mathrm{ext}}\right)}$$

where $C_{eq}^{*}$ is a dimensionless parameter and $C_{\mathrm{ini}},\;C_{eq},$ and $C_{ext}$ are the initial, equilibrium, and external gas concentrations (%), respectively. As per Table 1, $C_{eq}^{*}$ was lower for CO_2_ than for O_2_ for all the combinations of the tube diameter and length; therefore, the CO_2_ concentration took longer to reach equilibrium than the O_2_ concentration. These findings were in agreement with mass transfer coefficient *K*_CO_2__ being lower than *K*_O_2__ (Table 2). 

Table 1 also shows the time it took for the O_2_ and CO_2_ concentrations to achieve equilibrium for tubes of different dimensions. The longer the tube, the higher the airflow resistance, thus, the more time it required to achieve the equilibrium concentration. As expected, a tube with the length of 500 mm took longer time (compared with 50 mm and 250 mm tubes) to reach the equilibrium state regardless of the tube diameter. Increasing the tube diameter (at the length of 50 mm and 250 mm) from 2 mm to 4 mm reduced the time to reach equilibrium. A further increase of the diameter to 6 mm at the same lengths (50 mm and 250 mm) decreased the equilibrium time (Table 2; Figure 2). In general, a faster rate of exchange of gases was observed in the beginning due to a higher concentration difference between the inside and the outside of the box, which slowed down towards the end of the equilibrium point (Figure 2). The Reynolds number (Re) was calculated for tubes of all the diameters and found to be ˂2300 (1636, 817, and 545 for the tubes of 2 mm, 4 mm, and 6 mm in diameter, respectively) indicating that the airflow through the tubes was laminar. 

### 3.2. Mass Transfer Coefficient

**Figure 3:**
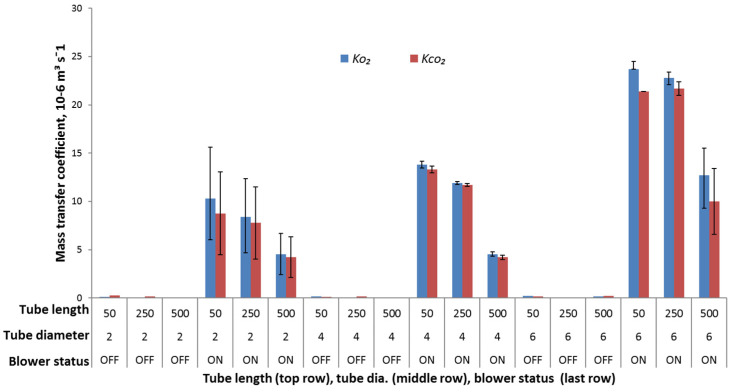
Forced airflow mass transfer coefficients *K*_O_2__ and *K*_CO_2__ as observed with different combinations of tube length and diameter with and without a mini air blower being ON (max. flow rate, 2.67 L min^−1^. Note: The error bars indicate the standard deviation of *K* values between the replicates.

Figure 3 shows the average gas diffusion rates (blower OFF) and forced airflow mass transfer coefficients (blower ON) obtained by the fit of the statistical model described by Equations (3) and (4) to the experimental data. The average permeability ratio (*β* = *K*_CO_2__*/K*_O_2__) at forced airflow was found to be 0.92 ± 0.06. This value is in agreement with the values as reported in the literature [8,9,12,13]. A comparison of the *K*_O_2__ and *K*_CO_2__ values obtained for tubes of different dimensions in the blower OFF (diffusion) and ON modes (forced airflow) is shown in Figure 3 and listed in Table 2. As observed in Table 2, during the initial 4 h period when the blower was OFF, the *K*_O_2__ values obtained at extreme boundary conditions of the tube of 500 mm L and 2 mm D and of 50 mm L and 6 mm D were 0.02 × 10^−6^ m^3^ s^−1^ and 0.23 × 10^−6^ m^3^ s^−1^, respectively. At the same extreme boundary conditions of the tube (L: 500 mm, D: 2 mm; L: 50 mm, D: 6 mm) the obtained *K*_CO_2__ values were 0.03 × 10^−6^ m^3^ s^−1^ and 0.17 × 10^−6^ m^3^ s^−1^, respectively. As expected, the *K*_O_2__ and *K*_CO_2__ values obtained were smaller in magnitude in comparison to the results published in the literature [9,11] due to the tubes being longer and thinner. During this 4 h period of air diffusion through the opening of the air blower, a 0.0186%/h increase in the O_2_ concentrations and a 0.0179%/h decrease in the CO_2_ concentrations were observed when it was OFF. This change was too small for a 190 L box. Therefore, it was evident that a tube alone cannot support the air exchange necessary for maintaining the desired gas composition inside the box, especially when fresh produce is stored in it. Using an air blower to induce forced airflow mass transfer resulted in a rapid increase in the *K*_O_2__ and *K*_CO_2__ values. The mass transfer coefficients due to the forced airflow at the extreme boundary conditions of the tube (L: 500 mm, D: 2 mm; L: 50 mm, D: 6 mm) were 4.55 × 10^−6^ m^3^ s^−1^ and 23.7 × 10^−6^ m^3^ s^−1^ for *K*_O_2__ and 4.35 × 10^−6^ m^3^ s^−1^ and 21.4 × 10^−6^ m^3^ s^−1^ for *K*_CO_2__. They were at least 100 times higher than diffusion. The higher the mass transfer coefficient, the faster the atmospheric gas change inside the storage box. ANOVA (*p* < 0.05) performed on the results obtained for the mass transfer coefficients due to forced airflow showed that increasing the tube length resulted in a significant decrease in the *K*_O_2__ and *K*_CO_2__ values, whereas increasing the tube diameter increased the *K*_O_2__ and *K*_CO_2__ values. The ANOVA (*p* < 0.05) results also showed that mass transfer coefficients varied more significantly with tube diameter than with tube length. The same trends were reported by Montanez, Rodríguez, Mahajan, and Frías [12] and by Silva, Chau, Brecht, and Sargent [9]. The sample coefficient of determination (*R*^2^) was found to be between 94.7% and 100% for all the combinations of tube D and L. 

In case of gas diffusion, the tubes of smaller diameter and longer length often resulted in slow gas exchange. The reason for this was larger gas diffusion resistance across the tube due to a small perforation area in comparison to long length of the tube. In this experiment, a tube of small diameter and long length was incorporated to limit the gas entering the storage box with sweet cherries when the air blower was OFF. Using this methodology, the authors eliminated the use of a valve that needs to be closed in an airtight manner in order to prevent leakage and control the desired gas concentration inside the storage box. A thin and long tube in this study itself reduced the leakage from the storage box, making it practically airtight. Addition of an air blower provided fast gas exchange (when switched ON), the frequency of switching whereof can be regulated and controlled as a function of temperature to maintain the desired gas composition. No studies have been reported in the literature that evaluated the convective mass transfer coefficient through a thin and long tube under modified atmosphere storage conditions. Data on the mass transfer coefficient are needed to model the storage system so that the ON frequency of the blower can be calculated in real time based on the temperature-dependent respiration rate.

### 3.3. Storage Trial with Sweet Cherries

**Figure 4:**
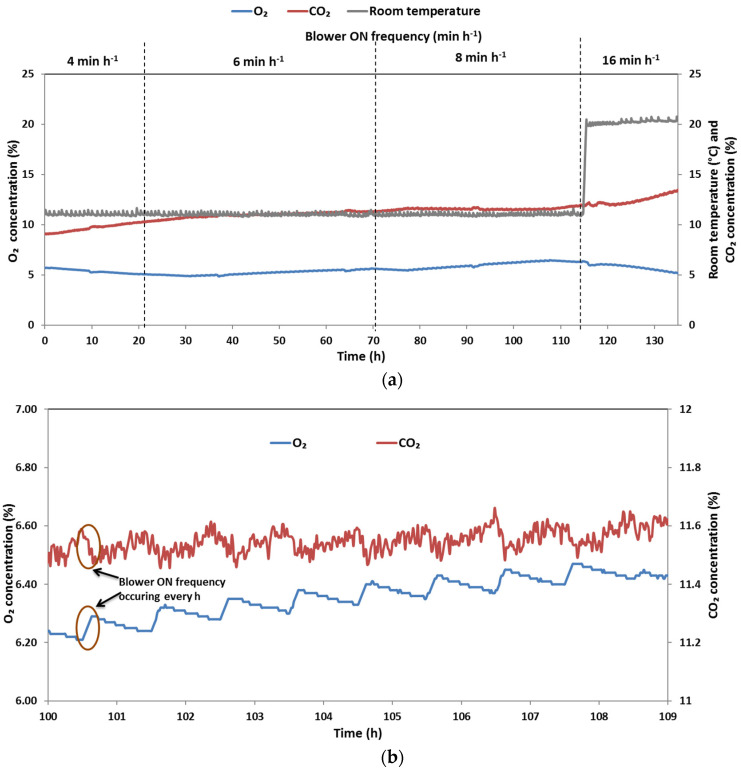
(**a**) Changes in the O_2_ and CO_2_ concentrations observed due to respiration of sweet cherries (25 kg) stored in a box (190 L) flushed with 6.5% O_2_ and 12.5% CO_2_ kept at 10 °C (until 114 h) and 20 °C (from 114 h to 135 h) and simultaneous active air flushing of the box achieved in real time through a tube (D: 4 mm, L: 250 mm) and a blower. (**b**) Closeup view of the gas concentration changes that occurred due to respiration of cherries (blower OFF) and forced air gas exchange (blower ON).

For storage trials with sweet cherries, choosing a tube length of 250 mm was beneficial over 500 mm (very slow gas exchange) and 50 mm (similar *K*_O_2__ and *K*_CO_2__ values when compared to 250 mm, but in this case a longer tube is preferred). In terms of the tube diameter, the 4 mm diameter was selected as the *K*_O_2__ and *K*_CO_2__ values were not significantly different between 4 mm and 6 mm. Figure 4a shows the changes in the concentrations of O_2_ and CO_2_ observed due to respiration of sweet cherries and intermittent forced airflow through the tube. The initial flushing of the metal box with modified atmosphere resulted in the gas composition of 5.7% O_2_ and 9% CO_2_. This initial flushing reduces the time required to achieve the equilibrium gas composition, thereby controlling the gas composition using the miniature air blower ON and OFF modes. Some previous works reported in the literature took between 3 d and 9 d to achieve the target modified gas concentrations inside the storage containers [18,21]. Another advantage of the initial flushing of the storage box with modified gas is that the respiration rate of the stored produce will be reduced since the beginning of storage period. This is beneficial for long-term storage and shelf life extension of stored produce. Initially, when the blower ON frequency was set to 4 min h^−1^, the concentrations of O_2_ and CO_2_ continued to decrease and increase, respectively. To maintain the gas concentrations inside the storage box within the set limits, the blower ON frequency was increased to 6 min h^−1^ at the 21st h. As a result, the O_2_ concentration increased and reached close to the initial concentration (5.7%). At the 70.5th hour, a slight decrease in the O_2_ concentration was observed, which was balanced by increasing the blower ON frequency to 8 min h^−1^. Increasing the blower ON frequency resulted in an increase in the O_2_ concentration to the maximum of 6.5%, which was initially the setpoint concentration. The CO_2_ concentration, however, remained constant between 11.2% and 11.9%, which was still within the desired window (10–15%). At the 117th hour, the storage temperature was changed from 10 °C to 20 °C. For every 10 °C increase in temperature, the respiration rate of fruit and vegetables generally increases two- or threefold within the range of temperatures normally encountered in the distribution and marketing chain [2,22]. Therefore, to compensate for the expected increase in the respiration rate of cherries, the blower ON frequency was doubled to 16 min h^−1^. As observed in Figure 4, at a higher temperature (20 °C), an increase in the CO_2_ concentration and a decrease in the O_2_ concentration were observed; however, they were still maintained within the recommended window. Figure 4b shows the closeup view of the O_2_ and CO_2_ changes that occurred due to respiration of cherries and gas exchange when the blower was switched ON. The graph clearly depicts the trend in changes of the O_2_ and CO_2_ concentrations in the blower ON (forced airflow between the inside and the outside of the metal box) and OFF modes (respiration of cherries). These data transferred every minute to a PC by RMS88 aided in real-time decision-making in terms of changing the blower ON frequency. Use of such a compact and modular system for automatic data collection and wireless transmission for real-time monitoring was useful in determining the stored fresh produce behavior, especially without missing any data due to delays with the possibility of automation [23]. Further, the authors recommend a blower ON frequency of 6 min h^−1^ and 8 min h^−1^ suitable for sweet cherries stored at 10 °C. At a higher temperature though, the gas concentration did not deviate too much from the recommended limit; however, a further study is recommended to investigate a more suitable blower ON frequency for sweet cherries.

Overall, the developed temperature-tunable active control system that uses a thin and long tube, a mini blower for forced airflow, and a modular sensor system, RMS88, for real-time measurement was useful in maintaining the desired gas composition for cherries. The application of the same system to different fresh F&V needs to be analyzed in further research. Due to the manual adjustment of the blower ON frequency, the O_2_ and CO_2_ concentrations could deviate from the given setpoint. Therefore, it is suggested to design the automatic control strategy that sets the blower ON frequency based on temperature. Further studies are required to develop the mathematical model that can consider selection of tube dimensions, amount of fresh produce, size of the storage chamber, temperature-dependent respiration rate and a correlation between the respiration rate and the blower ON time. Such a model will be useful for developing and optimizing a control strategy for automatic control of O_2_ and CO_2_ in the storage box.

## 4. Conclusions

In this study, a storage box with a thin and long tube and an air blower for inducing forced airflow was demonstrated to control the O_2_ and CO_2_ concentrations under variable temperatures. The storage box was able to maintain the desired gas concentrations within the set limits through the interplay of the natural gas modification due to the respiration rate of cherries and convective airflow through the tube. In comparison to a typical perforation-mediated storage box where passive air exchange takes place, addition of a miniature air blower for forced airflow resulted in an increase in the mass transfer coefficient by at least 100 times in comparison with diffusion. The proposed storage box was able to maintain O_2_ and CO_2_ within the set limits for sweet cherries under varying storage temperature. The modular sensor-based real-time O_2_ and CO_2_ measurement helped to manually control the blower ON frequency as a function of temperature. Such an MA storage box with a simple operation of airflow through a tube will be useful for storage and transportation of fresh produce. However, further efforts are needed to understand the influence of time, temperature, O_2_, CO_2_, ripening stage, and the initial microbial load on the respiration rate and how to integrate this in the mathematical model that predicts the blower operation time for given fresh produce. 

## Figures and Tables

**Table 1:** Equilibrium parameters $C_{eq}^{*}$ (O_2_) and $C_{eq}^{*}$ (CO_2_) calculated for tubes of different dimensions.

	$C_{eq}^{*}(O_{2}),\;\mathrm{Dimensionless}$			$C_{eq}^{*}\;({\mathrm{CO}}_{2}),\;\mathrm{Dimensionless}$		
Diameter/Length	50 mm	250 mm	500 mm	50 mm	250 mm	500 mm
2 mm	0.070	0.069	0.068	0.038	0.040	0.035
	(18.3) **	(22.3)	(37.1)	(21.9)	(23.4)	(39.9)
4 mm	0.069	0.069	0.067	0.039	0.037	0.037
	(15.8)	(17.6)	(36.7)	(15.6)	(17.7)	(40.3)
6 mm	0.069	0.069	0.070	0.039	0.033	0.034
	(10.4)	(10.8)	(20.3)	(10.9)	(11.7)	(26.5)

** Values in brackets indicate the time (h) it took for the O_2_ and CO_2_ concentrations to achieve the equilibrium state at the respective tube dimensions.

**Table 2:** Mass transfer coefficients (*K*_O_2__ and *K*_CO_2__) obtained at blower ON (forced airflow) and OFF (diffusion) for tubes of different dimensions.

Tube Dimension		*K*_O_2__ × 10^−6^ (m^3^ s^−1^)		*K*_CO_2__ × 10^−6^ (m^3^ s^−1^)	
D (mm)	L (mm)	OFF	ON	OFF	ON
2	50	0.11 ± 0.003 *	10.30 ± 0.03	0.29 ± 0.02	8.76 ± 0.01
2	250	0.08 ± 0.004	8.40 ± 0.004	0.15 ± 0.014	7.77 ± 0.01
2	500	0.02 ± 0.001	4.55 ± 0.001	0.03 ± 0.001	4.35 ± 0.002
4	50	0.14 ± 0.001	13.81 ± 0.01	0.11 ± 0.003	13.32 ± 0.01
4	250	0.07 ± 0.001	11.92 ± 0.01	0.17 ± 0.007	11.72 ± 0.02
4	500	0.02 ± 0.001	4.54 ± 0.002	0.01 ± 0.002	4.22 ± 0.003
6	50	0.23 ± 0.001	23.7 ± 0.08	0.17 ± 0.004	21.4 ± 0.058
6	250	0.02 ± 0.001	22.8 ± 0.060	0.01 ± 0.001	21.7 ± 0.055
6	500	0.18 ± 0.003	12.7 ± 0.060	0.23 ± 0.04	10 ± 0.054

* Values after “±” indicate the standard error of the estimates obtained with the least squares method in Statistica.

## Data Availability

The datasets generated for this study are available on request to the corresponding author.
